# Supplementary material for: Characterization of the Signaling Modalities of Prostaglandin E2 Receptors EP2 and EP4 Reveals Crosstalk and a Role for Microtubules
Source: Front Immunol. 2021 Feb 12;11:613286. doi: 10.3389/fimmu.2020.613286 (PMC7907432; doi:10.3389/fimmu.2020.613286)
Supplement: Supplementary file 1 [file Image_1.pdf]

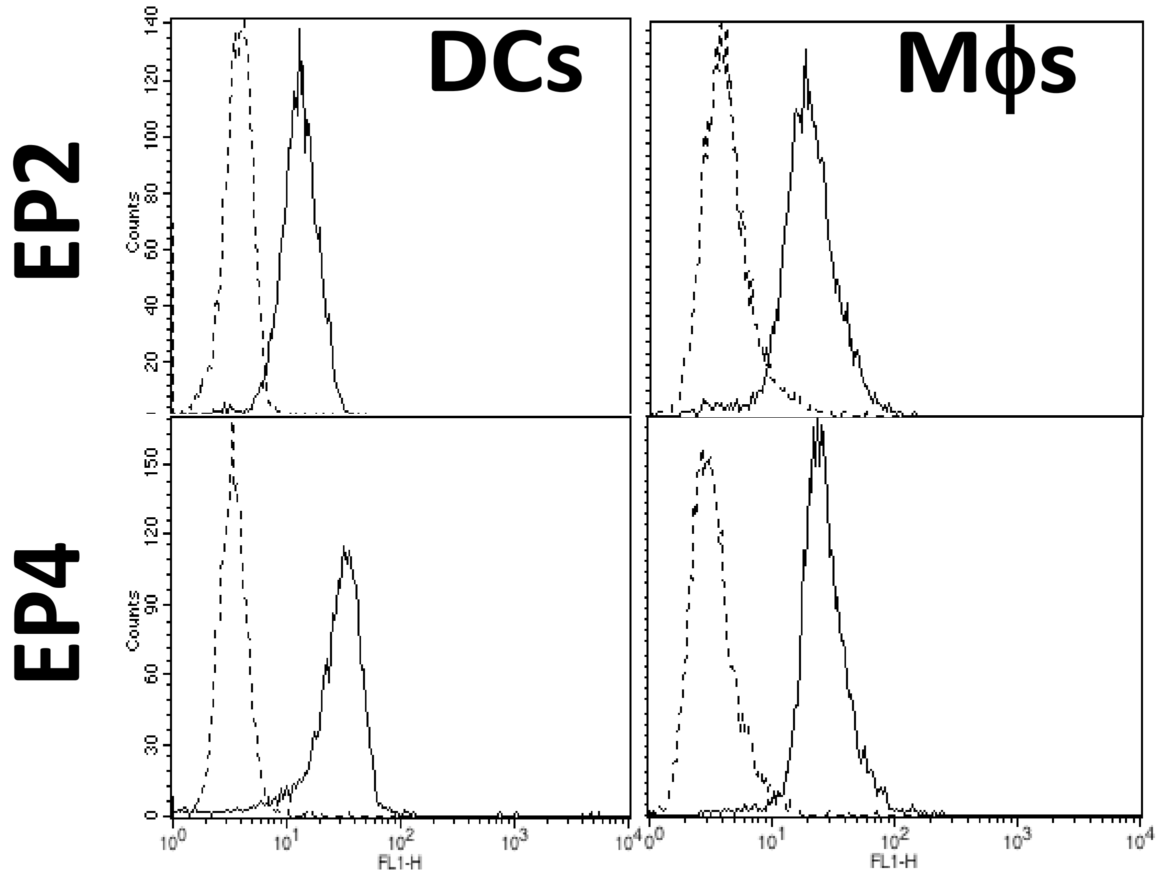

Supplementary figure 1: EP2 and EP4 cell surface expression. For flow cytometry analysis, dendritic cells (DCs) and RAW macrophages (MΦs) were labeled with anti-EP4 (C4, Santa Cruz) and anti-EP2 (H75, Santa Cruz) antibodies (5 µg/ml) in PBS, 0.5% BSA, and 0.01% sodium azide (30 min, 4°C), followed by incubation with Alexa-labeled secondary antibody (Invitrogen) for 30 min at 4°C. The fluorescence intensity was measured relative to isotype controls on a BD FACSCalibur.
