# Supplementary material for: Characterization of the Signaling Modalities of Prostaglandin E2 Receptors EP2 and EP4 Reveals Crosstalk and a Role for Microtubules
Source: Front Immunol. 2021 Feb 12;11:613286. doi: 10.3389/fimmu.2020.613286 (PMC7907432; doi:10.3389/fimmu.2020.613286)
Supplement: Supplementary file 2 [file Image_2.pdf]

(A)

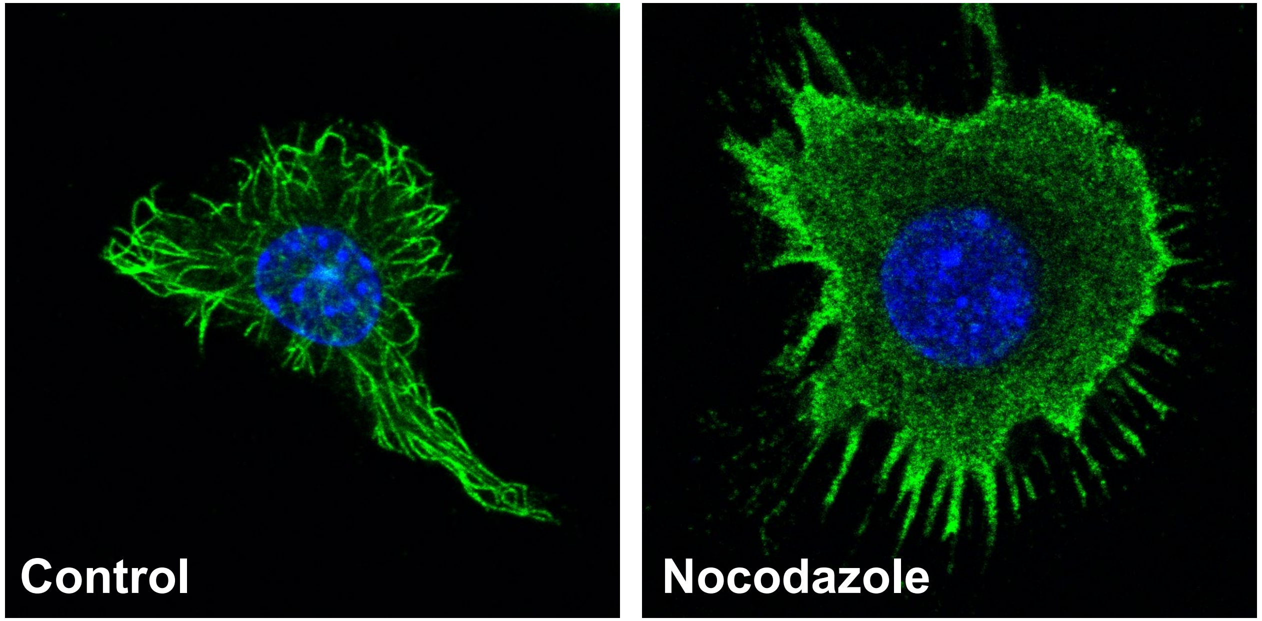

(B)

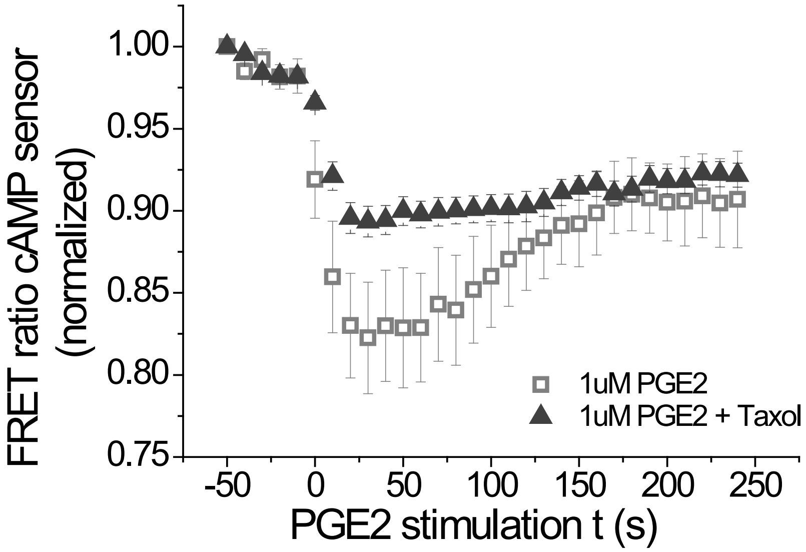

Supplementary Figure 2: Compounds altering microtubule structure and dynamics affect cAMP production. (A) Representative confocal images of cells left untreated (Control) or stimulated with 5 µM Nocodazole for 20 min and then quickly fixed and permeabilized. Microbutules (green) are labelled with a mouse anti-tubulin (clone E7, supernatant) and the nucleus (blue) is labelled with DAPI. (B) The FRET ratio of t-Epac-vv was measured in cells that were untreated or pretreated with 5 µM Taxol for 20 mins before and after addition of 1 µM PGE2.
